# IRES–cargo interplay structurally modulates circular RNA translation

**DOI:** 10.1038/s41422-026-01233-9

**Published:** 2026-02-26

**Authors:** Youkui Huang, Yao-Qi Chen, Si-Yu Lou, Xiang Gao, Yu-Xin Liu, Yu-Lu Zhang, Fang Nan, Ling-Ling Chen, Li Yang

**Affiliations:** 1 State Key Laboratory of RNA Innovation, Science and Engineering, New Cornerstone Science Laboratory, CAS Center for Excellence in Molecular Cell Science, Shanghai Institute of Biochemistry and Cell Biology, University of Chinese Academy of Sciences, Chinese Academy of Sciences, Shanghai, China; 2 Center for Molecular Medicine, Children’s Hospital of Fudan University and Shanghai Key Laboratory of Medical Epigenetics, International Laboratory of Medical Epigenetics and Metabolism, Ministry of Science and Technology, Institutes of Biomedical Sciences, Fudan University, Shanghai, China; 3 Key Laboratory of Systems Health Science of Zhejiang Province, School of Life Science, Hangzhou Institute for Advanced Study, University of Chinese Academy of Sciences, Hangzhou, Zhejiang China; 4 School of Life Science and Biotechnology, Shanghai Jiao Tong University, Shanghai, China; 5 School of Life Science and Technology, ShanghaiTech University, Shanghai, China; 6 Shanghai Academy of Natural Sciences (SANS), Shanghai, China

**Keywords:** Translation, Cell biology

Dear Editor,

Recent studies on endogenous circular RNAs (circRNAs) have revealed their high stability and unique conformation.^1–3^ Engineered in vitro circularized RNAs (ivcRNAs), which serve as advanced platforms for sustained protein expression,^4,5^ exhibit greater stability and lower immunogenicity than their linear counterparts.^4–8^ Because ivcRNAs lack a 5′ *m⁷G* cap structure, they typically rely on a structured internal ribosome entry site (IRES) to initiate cap-independent translation, a process that depends on the distinct secondary and tertiary structures of viral IRESs. However, whether — and to what extent — cargo sequences within the ivcRNA platform affect IRES structure and translational efficiency remains poorly understood. Here, we demonstrate that extensive interactions between the Salivirus A IRES (*SV-A*) and certain cargo sequences compromise IRES structural integrity, thereby impairing translation. Correspondingly, disrupting unfavorable *SV-A*–cargo pairing restores efficient ivcRNA translation. To evaluate functional IRES elements for circular RNA translation, we engineered viral IRESs together with a firefly *luciferase* (*fLuc*) cargo sequence into the *Anabaena* transfer RNA (tRNA)^Leu^-derived 27-nt permuted intron–exon (PIE) system (Ana_PIE_27 nt; Fig. 1a).^7^ In total, 45 IRES elements derived from the 5′ untranslated regions (UTRs) of various viruses were examined,^9,10^ and were theoretically categorized into five types based on sequence length, homology, and secondary structure (Fig. 1b; Supplementary information, Table S1). Prior to transfection into various cell types for translation analysis, each ivcRNA was generated by in vitro transcription and self-catalytic circularization, and subsequently validated by denaturing urea–PAGE (polyacrylamide gel electrophoresis) (Supplementary information, Fig. S1). Using the *fLuc–nLuc* reporter assay (Fig. 1a), type V IRESs generally exhibited superior translation efficiency (Fig. 1c). Specifically, translation driven by type V IRESs was significantly higher than that of types I/II/III/IV in HeLa and C2C12 cells, and higher than that of types III/IV in 293FT and Huh-7 cells (Fig. 1c). Notably, the type V *SV-A* displayed overall higher translation efficiency than the commonly used type I Coxsackievirus B3 IRES (*CVB3*),^4,11,12^ although translation efficiency varied across the tested cell lines (Fig. 1c).

**Fig. 1:**
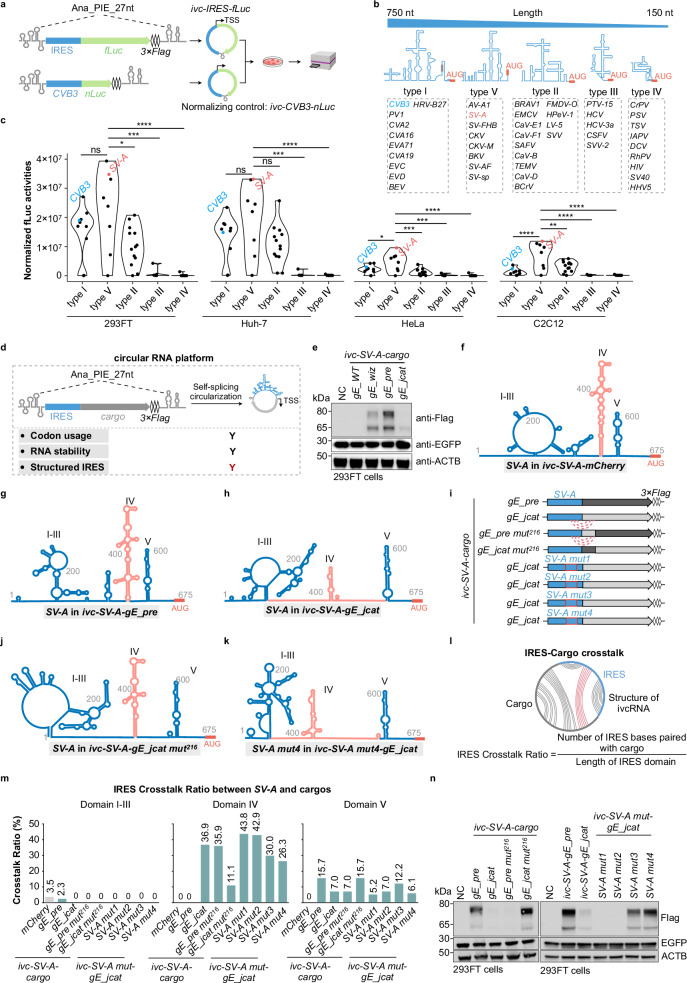
IRES–cargo interactions in ivcRNAs hinder the translational efficiency of *SV-A*-driven cargo sequences. **a**–**c** Screening of functional IRESs for ivcRNA translation. Using the fLuc reporter assay (**a**), different types of IRESs (**b**) were screened for their ability to drive ivcRNA translation (**c**). Statistical significance was evaluated by one-way ANOVA with Tukey’s post hoc test. ns not significant (*P* > 0.05; *P < 0.05; **P < 0.01; ***P < 0.001; ****P < 0.0001). **d** Schematic illustrating key factors for sequence optimization in the ivcRNA translation platform. TSS, translation start site. Y, yes; red Y indicates a newly introduced parameter for ivcRNA design. **e** Western blot analysis of codon-optimized *gE* expression from *SV-A*-driven ivcRNAs. **f**–**h** CircSHAPE-guided secondary structure models of *SV-A* in ivcRNAs carrying *mCherry* (**f**), *gE_pre* (**g**), or *gE_jcat* (**h**). **i**–**n** Restoration of *SV-A* domain IV structure and gE expression by disrupting IRES–cargo base pairing. Secondary structures of *SV-A* in various engineered ivcRNAs (**i**) were modeled for *ivc-SV-A-gE_jcat mut*^*216*^ (**j**) and *ivc-SV-A mut4-gE_jcat* (**k**). Computational analysis of the IRES Crosstalk Ratio (**l**) revealed changes in *SV-A*–cargo interactions (**m**). Western blot analysis detected gE protein expression from various engineered ivcRNAs in 293FT cells (**n**). mut, mutant. See Supplementary information, Fig. S6, for *SV-A* structures in additional ivcRNAs. In **e**, **n**, NC indicates the negative control (transfected with *EGFP* mRNA only). In **i**, dotted lines denote IRES–cargo interactions, and red boxes highlight mutated regions within the IRES. In **f**, **g**, **h**, **j**, **k**, domain IV is indicated in magenta.

To further assess the translational capacity of the type V *SV-A*-driven ivcRNA platform, *SV-A* was fused with several wild-type (WT) cargo sequences encoding mCherry; the capsid protein VP2 of Senecavirus A; antigen 85A (Ag85A) of *Mycobacterium tuberculosis*; and a carboxyl-terminally truncated envelope glycoprotein E (gE) of Varicella–Zoster virus (Supplementary information, Fig. S2a and Table S1). These ivcRNAs were prepared and quality-validated as described above (Supplementary information, Fig. S2a, b). Western blot analysis showed that *SV-A*-driven *mCherry* yielded the highest translation level, followed by moderate expression of VP2 and Ag85A, whereas gE protein was barely detectable (Supplementary information, Fig. S2c). Notably, endogenous *β-actin* (*ACTB*) and the transfection control *EGFP* mRNA were efficiently translated. Because circular RNA translation efficiency is influenced by codon usage, RNA stability, and IRES structure (Fig. 1d), we first optimized the codon usage of *gE_WT* to enhance protein expression. Three algorithms were applied to generate codon-optimized *gE* sequences (*gE_wiz*, *gE_pre*, and *gE_jcat*; see Supplementary information, Methods, and Table S1). These variants shared ~72%–74% sequence identity with *gE_WT* but exhibited higher codon adaptation index (CAI) values (Supplementary information, Fig. S2d, e). A new set of *SV-A*-driven ivcRNAs harboring these optimized sequences was validated prior to downstream analyses (Supplementary information, Fig. S2f). Intriguingly, *gE_jcat* showed unexpectedly lower *SV-A*-driven translation in the ivcRNA context compared with *gE_wiz* and *gE_pre* (Fig. 1e). This impaired gE expression was unlikely due to reduced translational capacity or RNA instability, as *gE_jcat* was efficiently translated in a linear mRNA context and exhibited comparable RNA abundance to the other codon-optimized *gE* variants in 293FT cells (Supplementary information, Fig. S2g–i).

We next hypothesized that different codon-optimized *gE* cargo sequences might affect *SV-A* IRES folding and structure within the circular RNA, thereby modulating ivcRNA translation. To test this hypothesis, we first predicted secondary structures of long ivcRNAs, including the well-translated *ivc-SV-A-mCherry* (1482 nt) and *ivc-SV-A-gE_pre* (2490 nt), using the RNAfold algorithm (http://rna.tbi.univie.ac.at/cgi-bin/RNAWebSuite/RNAfold.cgi). In contrast to the published *SV-A* structure (Supplementary information, Fig. S3a),^13^ these sequence-only secondary structure predictions for *SV-A* exhibited substantial deviations (Supplementary information, Fig. S3b, c), highlighting the limitations of purely computational structure prediction for long circular RNAs. To overcome this limitation, we determined the in-cell structure of ivcRNAs using SHAPE-MaP (selective 2′-hydroxyl acylation analyzed by primer extension and mutational profiling), and incorporated the resulting SHAPE reactivities into RNAfold to model secondary structures (circSHAPE-MaP; Supplementary information, Fig. S3d; Materials and Methods; Tables S2 and S3). These predicted structural models indicated that the *SV-A* structures in *ivc-SV-A-mCherry*and *ivc-SV-A-gE_pre* closely resembled the previously reported *SV-A* model (Supplementary information, Fig. S3a), as evidenced by preservation of the cruciform domain IV (Fig. 1f, g; Supplementary information, Figs. S3e and S4b). In contrast, notable structural discrepancies in *SV-A* were observed among the *ivc-SV-A-gE* variants (Fig. 1g, h; Supplementary information, Fig. S4). Specifically, *SV-A* domainIV formed base-pairing interactions with the *gE_wiz* or *gE_jcat* cargo sequences in ivcRNAs (Supplementary information, Fig. S4a, c), leading to disruption of the *SV-A* domain IV structure, most prominently in the *ivc-SV-A-gE_jcat* platform (Fig. 1h; Supplementary information, Fig. S4c).

Detailed structural analysis revealed that the 676–891 nt region within *ivc-gE_jcat* (corresponding to the 1–216 nt region within *gE_jcat*) interacted with *SV-A* domain IV (the 368–417 nt and 502–550 nt regions) of *ivc-SV-A-gE_jcat* (Supplementary information, Fig. S4c). We therefore proposed that disrupting this specific interaction could restore *SV-A* folding and *SV-A*-driven translational capacity. To test this hypothesis, we swapped the 1–216 nt region between the *gE_pre* and *gE_jcat* sequences to generate *ivc-SV-A-gE_pre mut*^*216*^ and *ivc-SV-A-gE_jcat mut*^*216*^. In parallel, naturally occurring mutations were introduced into *SV-A* domain IV to generate corresponding *gE_jcat* ivcRNAs driven by different *SV-A* mutants (Fig. 1i; Supplementary information, Fig. S5 and Table S1). In-cell circSHAPE-MaP analysis revealed that subtle sequence alterations (< 20 nt in each of the cargos or the *SV-A* mutants) were sufficient to reciprocally remodel base-pairing interactions between *SV-A* domain IV and the *gE_jcat* sequence (Supplementary information, Figs. S5b, c and S6a). Specifically, the cruciform structure of domain IV  was reversed in *ivc-SV-A-gE_pre mut*^*216*^ and *ivc-SV-A-gE_jcat mut*^*216*^, and partially restored in *ivc-SV-A mut3-gE_jcat* and *ivc-SV-A mut4-gE_jcat* (Fig. 1g, h, j, k; Supplementary information, Fig. S6b–e).

To quantitatively assess IRES structural integrity across different ivcRNAs, we employed two metrics: the IRES Crosstalk Ratio, which quantifies IRES bases paired with the cargo sequence, and the IRES Structure Consistency, which measures IRES bases consistent with the IRES structure in *ivc-IRES-mCherry* (Fig. 1l; Supplementary information, Fig. S7a). Accordingly, *ivc-SV-A-gE_jcat mut*^*216*^, *ivc-SV-A mut3-gE_jcat*, and *ivc-SV-A mut4-gE_jcat* exhibited higher IRES Structure Consistency and lower IRES Crosstalk Ratio — particularly within domain IV — than *ivc-SV-A-gE_jcat*, *ivc-SV-A mut1-gE_jcat*, and *ivc-SV-A mut2-gE_jcat* (Fig. 1m; Supplementary information, Fig. S7b). As expected, gE expression was fully restored in *ivc-SV-A-gE_jcat mut*^*216*^ and partially recovered in *ivc-SV-A mut3-gE_jcat* and *ivc-SV-A mut4-gE_jcat*, but remained suppressed in *ivc-SV-A mut1-gE_jcat* and *ivc-SV-A mut2-gE_jcat* across multiple cell types (Fig. 1n; Supplementary information, Fig. S7c, d). Importantly, gE protein expression from the various ivcRNAs strongly correlated with both the IRES Crosstalk Ratio and the IRES Structure Consistency, specifically within domain IV of the *SV-A* IRES (Supplementary information, Fig. S7e, f and Table S4). Collectively, these results support our hypothesis that IRES–cargo interactions compromise IRES structural integrity, thereby impairing cap-independent translation in ivcRNAs driven by the corresponding IRESs.

The identification of IRES–cargo interactions and their effects on ivcRNA translation highlights the importance of understanding IRES folding dynamics and structural integrity within the circular RNA conformation, which is distinct from that of linear mRNAs, for the rational design of translatable ivcRNAs. Given that distinct gE protein expression levels were observed from different combinations of cargo sequences and IRESs (Fig. 1e; Supplementary information, Fig. S8), we infer that IRES–cargo interactions generally influence downstream protein translation, likely through differential structural interference with the IRES. Taken together, our findings demonstrate that IRES–cargo crosstalk affecting IRES structural consistency and ivcRNA translation is critical for the rational design of therapeutic ivcRNAs for protein expression. Future research is warranted to establish generalized ivcRNA design principles that incorporate IRES–cargo interplay, cargo secondary structure, and codon usage to achieve desired protein expression outcomes.

## Supplementary information

Supplementary information

Supplementary information, Table S1

Supplementary information, Table S2

Supplementary information, Table S3

## Data Availability

The RNA sequencing data for circSHAPE-MaP can be accessed from the National Center for Biotechnology Information (NCBI) under BioProject ID: PRJNA1419701.
