# Supplementary material for: IRES–cargo interplay structurally modulates circular RNA translation
Source: Cell Res. 2026 Feb 26;36(5):377–80. doi: 10.1038/s41422-026-01233-9 (PMC13092624; doi:10.1038/s41422-026-01233-9)
Supplement: Supplementary file 1 — Supplementary information [file 41422_2026_1233_MOESM1_ESM.pdf]

Supplementary information, Materials and Methods, Figures and Tables

IRES-cargo interplay structurally modulates circular RNA translation

Youkui Huang[1,7], Yao-Qi Chen[2,7], Si-Yu Lou[1,7], Xiang Gao[3,7], Yu-Xin Liu[1], Yu-Lu Zhang[3], Fang Nan[1,2], Ling-Ling Chen[1,4,5,6*], Li Yang[2*]

Materials and Methods

Cell culture

Human cell lines including HeLa and U-2 OS cells were purchased from the American Type Culture Collection (ATCC; http://www.atcc.org); Huh-7 was purchased from National Collection of Authenticated Cell Cultures; 293FT was purchased from ThermoFisher. Mouse cell line, C2C12 cells, was purchased from the ATCC. The 293FT, Huh-7, C2C12, Hela and U-2 OS cells were maintained in DMEM, and all of them were supplemented with 10% FBS and 0.1% penicillin–streptomycin. We cultured cell lines at 37 °C in a 5% CO_2 cell-culture incubator and tested all cell lines routinely to exclude mycoplasma contamination.

Plasmid constructions

To construct in *vitro* transcription template of ivcRNA, IRES sequences and protein coding sequences were individually synthesized from GenScript (Nanjing, China), and insert into the Ana_PIE_27nt backbone plasmid.

Several wide type cargo sequences were used in this study, including the *VP2* of Senecavirus A[1], the *Ag85A* of Mycobacterium tuberculosis[2], and the carboxyl-terminal truncated *gE* of Varicella-Zoster Virus[3]. Three algorithms (Genewiz: https://climsprod.genewiz.com.cn/Toolbox/CodonOptimization; pre: a customized codon-optimization algorithm developed by RISE™ (RiboX Therapeutics, unpublished); JCat: https://www.jcat.de[4]) were adopted to design codon-optimized *gE* sequences, *gE_wiz*, *gE_pre* and *gE_jcat*. These algorithms employ distinct strategies for codon optimization. Genewiz, referred to as ‘wiz’ in this study for simplicity, replaces low-frequency codons with high-frequency synonyms while adjusting local GC content and repeat sequences to achieve high CAI values. JCat, referred to as ‘jcat’ in this study, pursues CAI maximization by uniformly assigning the single most frequent codon to each amino acid. A customized codon-optimization algorithm

developed by RISE™, referred to as 'pre' in this study, algorithm utilizes a more integrated approach, employing a sliding window and a multi-factor fitness function that simultaneously optimizes codon usage, GC content, and avoids repetitive sequences.

To express gE protein by plasmid, WT and optimized *gE* variants DNA sequences were cloned into the p23-phage vector. All constructs were confirmed by Sanger sequencing.

All related sequences are listed in Supplementary Table S1.

***In vitro* RNA transcription, circularization and purification**

In vitro transcription was performed as previously described with slight modifications.[5] Briefly, ivcRNA precursors were transcribed from 1 µg linearized plasmid using RiboMAX large scale RNA production system (Promega) according to the manufacturer's protocol. After DNA template digestion, transcribed RNAs were recovered using the MEGAclear Transcription Clean-up kit (Invitrogen). For *in vitro* circularization, transcribed RNAs were heated to 70°C for 5 min and immediately placed on ice for 2 min, then the circularization was catalyzed for 8 min at 55°C in 1×T4 RNA ligase buffer (NEB) supplemented with 2 mM GTP, followed by column purification. To further enrich ivcRNAs, 20 U RNase R was added to 100 µg RNA mixture for 3 h at 37°C. Finally, ivcRNAs was column purified and checked in 4% denaturing urea-PAGE gel, with the Ambion® RNA Millennium^{TM} (Invitrogen) as the molecular weight marker.

Cell transfection

RNA transfection was performed using Lipofectamine MessengerMax reagent (ThermoFisher) according to the manufacturer's protocols. To evaluate protein expression level of ivcRNAs, cells were seeded in a 24-well plate at a density of 2 ×

10^5 cells per well and transfected with 100 ng ivcRNAs per well. 10 ng *ivc-CVB3-nLuc* or *EGFP* mRNA was co-transfected as internal control. After 24 h of transfection, cells were collected for further analysis.

Luciferase assay

Cells were harvested in 200 μL 1× Passive Lysis Buffer (Promega) for 15 minutes rotation at room temperature, and centrifuged at 10,000g for 1 minutes to collect supernatant. Luciferase activity was detected using Nano-Glo Dual-Luciferase Reporter Assay System (Promega) according to manufacturer's protocol. Briefly, 10 μL supernatant was added to a 384-well white-bottom plate (PerkinElmer). Then 10 μL ONE-Glo EX substrate was added to each well and mix well by centrifugation at 1,000 rpm for 2 minutes. Firefly Luciferase (fLuc) activity was quantified with PerkinElemer EnVision instrument. For nano Luciferase (nLuc) luminescence quantification, 10 μL NanoDLR Stop & Glo Reagent was added followed by centrifugation at 1,000 rpm for 2 minutes. Then nLuc luminescence was measured after incubation for 10 minutes. Relative firefly luciferase activity was calculated by normalizing the nLuc signal to that in mock well.

Western blotting

Cells were collected and resuspended in lysis buffer (50 mM Tris pH 8.0, 1% NP-40, 0.5% sodium deoxycholate, 0.1% SDS, 150 mM NaCl, and 1× cocktail) for 10 min on ice, followed by centrifugation at 1,2000 g for 10 min. Subsequently, supernatants were resolved on polyacrylamide gel with 10% SDS and analyzed by western blot with anti-gE (Santa Cruz Biotechnology, 1:500 dilution), anti-Flag (Sigma, 1:1,000 dilution), anti-EGFP (ThermoFisher, 1:1,000 dilution), or anti-ACTB (Sigma, 1:5,000 dilution) antibodies. Specifically, protein samples were separated on a 4–12% Bis-Tris gel using MOPS running buffer, with Thermo Scientific PageRuler Prestained Protein Ladder as

the molecular weight marker.

RNA isolation and RT-PCR

Total RNAs from cultured cells were extracted with Trizol (Invitrogen) according to the manufacturer's protocol, and then were reverse transcribed with SuperScript III (Invitrogen) to generate cDNAs. To quantify the mRNA levels of *gE_WT* and its codon-optimized variants expressed from the plasmid system, we used a universal primer pair for RT-qPCR analysis. For in cell RNA stability, abundance of each circle at different time points was determined by RT-qPCR with corresponding primers targeting IRESs.

Primers for RT-qPCR are listed in Supplementary Table S2.

circSHAPE-MaP

To determine the IRES structure in ivcRNAs, SHAPE probing was performed as described with modifications for circular RNA (circSHAPE-MaP).[6-9] For in cell RNA probing, 293FT cells were transfected with ivcRNAs. After 24 h, cells were incubated with 300 μL medium containing DMSO or 200 mM NAI (EMD Millipore) for 10 min at 37 °C. After washed with DPBS, RNAs were isolated with 1 ml Trizol reagent (Invitrogen) according to the manufacturer's protocol. To enrich ivcRNAs, 10 μg total RNA samples were treated with 20 U RNase R for 1 h at 37°C. After digestion, RNAs were recovered by RNAClean XP beads (Beckman). Isolated RNAs were processed to SHAPE-MaP reverse transcription by SuperScript II (Invitrogen) in buffer containing 6 mM Mn^{2+} and gene-specific primers. Target DNA amplification was performed with Taq DNA polymerase. The resulting PCR products were further isolated by agarose gel electrophoresis. For original and mutated *ivc-SV-A-gE_jcat* and *ivc-SV-A-gE_pre*, full-length of ivcRNA or fragments of IRES were amplified and sequenced (Fig. 1g, h, j, k; Supplementary Fig. S4b, c, S6b-e). For other ivcRNA, the fragments of IRES (including flanking 100 nt) were sequenced (Fig. 1f; Supplementary Fig. S3e, S4a). For

ivc-SV-A-gE_jcat and *ivc-SV-A-gE_pre*, *SV-A* structure modeling using SHAPE reactivity from the IRES region or full-length ivcRNA showed consistency.

circSHAPE-MaP libraries were prepared from 1 ng of each DNAs, and size-selected with Ampure XP beads (Beckman) with a 1.8:1 (bead to sample) ratio to obtain library DNA products spanning 100-400 bp in length. Final libraries were quantified using Agilent Bioanalyzer 2100 and QuBit high-sensitivity dsDNA assay and subjected to deep sequencing with Illumina NovaSeq 6000 system at Sequanta Technologies.

Primers for SHAPE-MaP reverse transcription and DNA amplification are listed in Supplementary Table S2. The SHAPE-MaP reactivity profiles are listed in Supplementary Table S3.

SHAPE reactivity calculation

Reads from circSHAPE-MaP sequencing data were processed according to CIRCshapemapper pipeline.[8,9] Firstly, the reads were subjected to remove primer using Cutadapt (V4.4), and then clean data were analyzed by ShapeMapper (V2.2) to calculate the SHAPE reactivity with the parameters (--name IRES-cargos –target ivc-IRES-cargo.fa –out IRES-cargos --verbose --nproc 3 --min-depth 100 --output-parsed-mutations --output-counted-mutations --modified --untreated --denatured). The reference sequences in this analysis were generated according to PCR primers.

RNA secondary structure modeling and visualization

All MFE secondary structures of ivcRNAs were modeled via RNAfold (V2.6.4) with or without SHAPE reactivities.[10] The sequence-only predicted structure was modeled with the parameters (--MEA -p -d2 -circ --maxBPspan=700) and SHAPE-guided structure was modeled with the parameters (--MEA -p -d2 --circ --shape --shapeMethod=Z --maxBPspan=700). All structures were visualized with StructureEditor. The base pair probability matrices were obtained from RNAfold output

files (RNA_dp.ps).

IRES Crosstalk Ratio

To quantify the extent of interaction between the IRES and the cargo sequences in ivcRNAs, the IRES Crosstalk Ratio was calculated by dividing the number of bases of the IRES paired with the cargo by the sequence length of the IRES.

IRES Structure Consistency

To assess the secondary structure consistency between sequence-only and SHAPE-guided structures, or the structures of ivcRNAs and the reference structures (*ivc-SV-A-mCherry*), we compared the pairing status of each nucleotide position in the IRES region. The consistency was determined as the number of bases with identical pairing status (including the same pairing bases and unpaired bases at the same position in both structures) divided by the IRES length.

Statistical analysis

Mean values, SD values, one-way ANOVA and Student's *t*-test (unpaired) were calculated using Prism software (GraphPad). ns, not significant $P > 0.05$, *$P < 0.05$, **$P < 0.01$, ***$P < 0.001$, ****$P < 0.0001$.

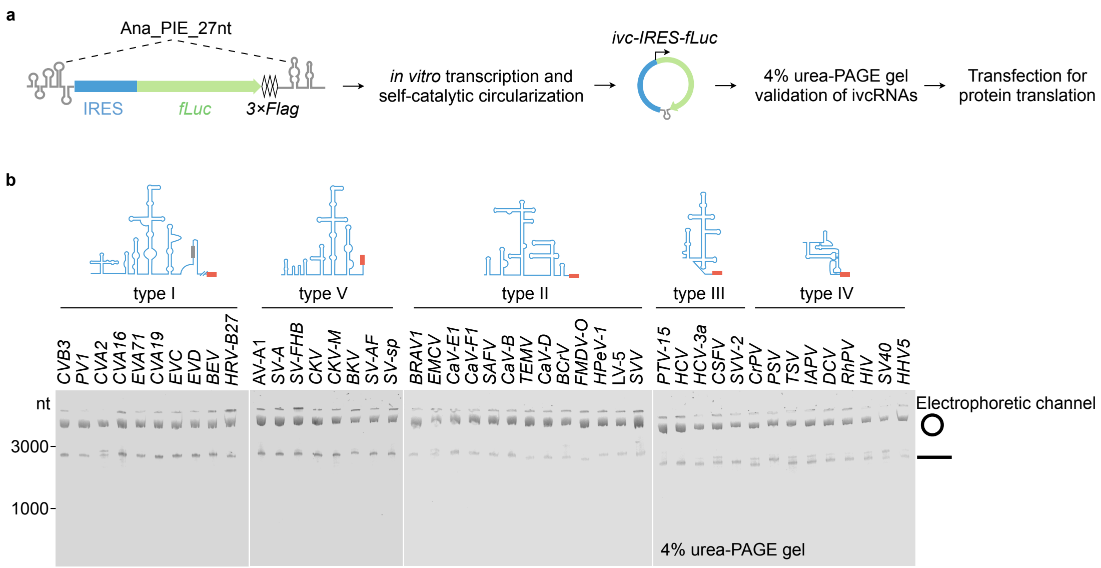

Supplementary Fig. S1: Characterization of IRES driven ivcRNA translation.

a, Schematic of IRES activity evaluation in ivcRNAs. *fLuc*-ivcRNAs containing various IRESs were *in vitro* synthesized using Ana_PIE_27nt system. After urea-PAGE gel validation, ivcRNAs were transfected into cells to evaluate IRES translation capability. fLuc, firefly Luciferase. PAGE, polyacrylamide gel electrophoresis.

b, *In vitro* synthesized ivcRNAs containing different IRESs were examined by 4% denaturing urea-PAGE gel. Top, secondary structure models of five types of IRESs. Bottom, validation of ivcRNAs containing different IRESs. Circular RNA is indicated by black circle, nicked RNA indicated by black line.

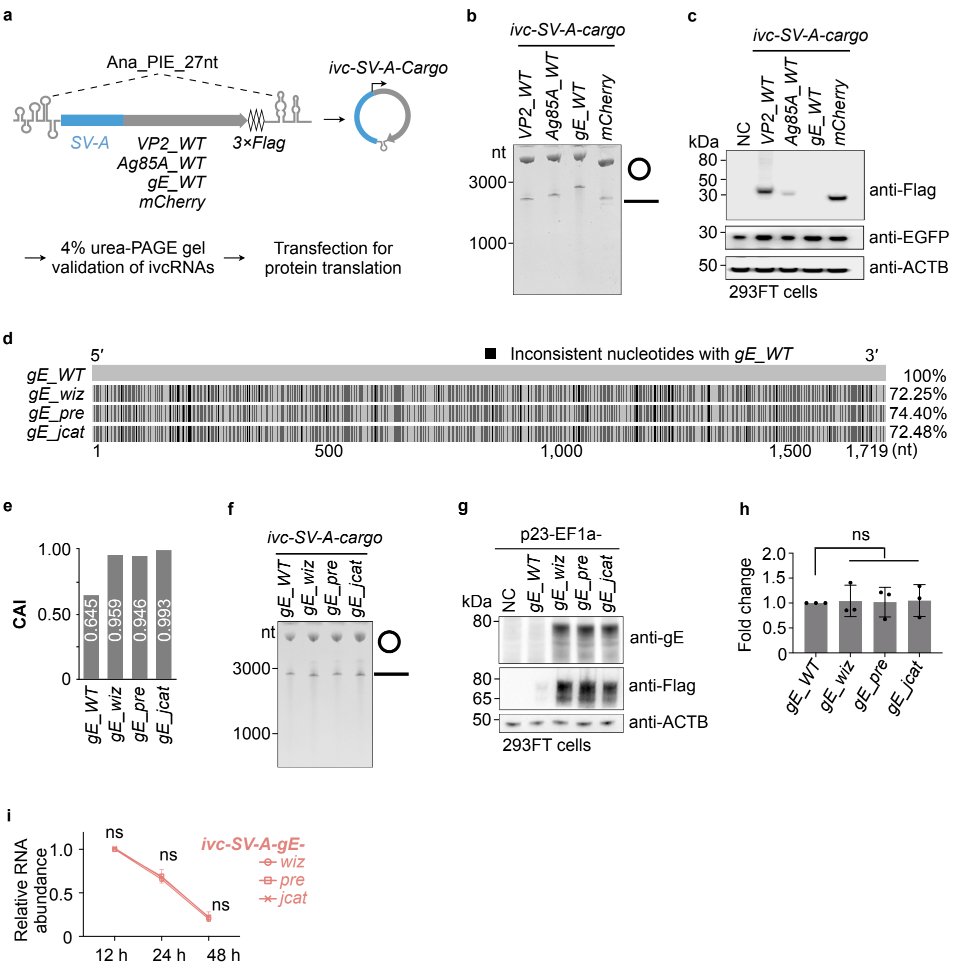

Supplementary Fig. S2: Codon optimization to improve gE protein expression in ivcRNAs.

a, Schematic of evaluation of *SV-A*-driven ivcRNA translation. Distinct ivcRNAs containing various cargos were *in vitro* synthesized using Ana_PIE_27nt system. WT, wild type.

b, *In vitro* synthesized ivcRNAs containing different cargos were examined by 4% denaturing urea-PAGE gel. Circular RNA is indicated by black circle, nicked RNA indicated by black line.

c, The cargo expression was detected by Western Blot (WB). The co-transfected *EGFP* mRNA was used as internal control. The β-Actin (ACTB) acted as endogenous protein control.

d, The optimized *gE* sequences were aligned to *gE_WT.* The inconsistent nucleotides

with *gE_WT* were labeled in black, and the sequence identity relative to *gE_WT* was shown on the right.

e, Improve CAI of optimized *gE* variants compared to *gE_WT* sequence. CAI, codon adaption index.

f, *In vitro* synthesized ivcRNAs containing *gE* variants were examined by 4% denaturing urea-PAGE gel. Circular RNA is indicated by black circle, nicked RNA indicated by black line.

g, Similar gE protein expression level from codon-optimized *gE* variants in p23 plasmid. Protein expression from codon-optimized *gE* sequences and *gE_WT* was detected by WB with anti-Flag and anti-gE antibody respectively. Condon optimization enhances gE protein expression from codon-optimized *gE* variants compared to *gE_WT* after transfected with p23-EF1α plasmids. NC, negative control, transfected with empty vector.

h, RT-qPCR analysis revealed comparable mRNA expression levels between *gE_WT* and its codon-optimized variants following transfection of the plasmid constructs into 293FT cells. Statistical significance was evaluated by one-way ANOVA. ns, not significant, $P > 0.05$.

i, Similar RNA stability of *gE* ivcRNAs after transfected into 293FT cells. Relative RNA abundance at each time point (0 h, 24 h and 48 h) of *gE* ivcRNAs was evaluated by qRT-PCR. ns, not significant, $P > 0.05$, student's *t*-test.

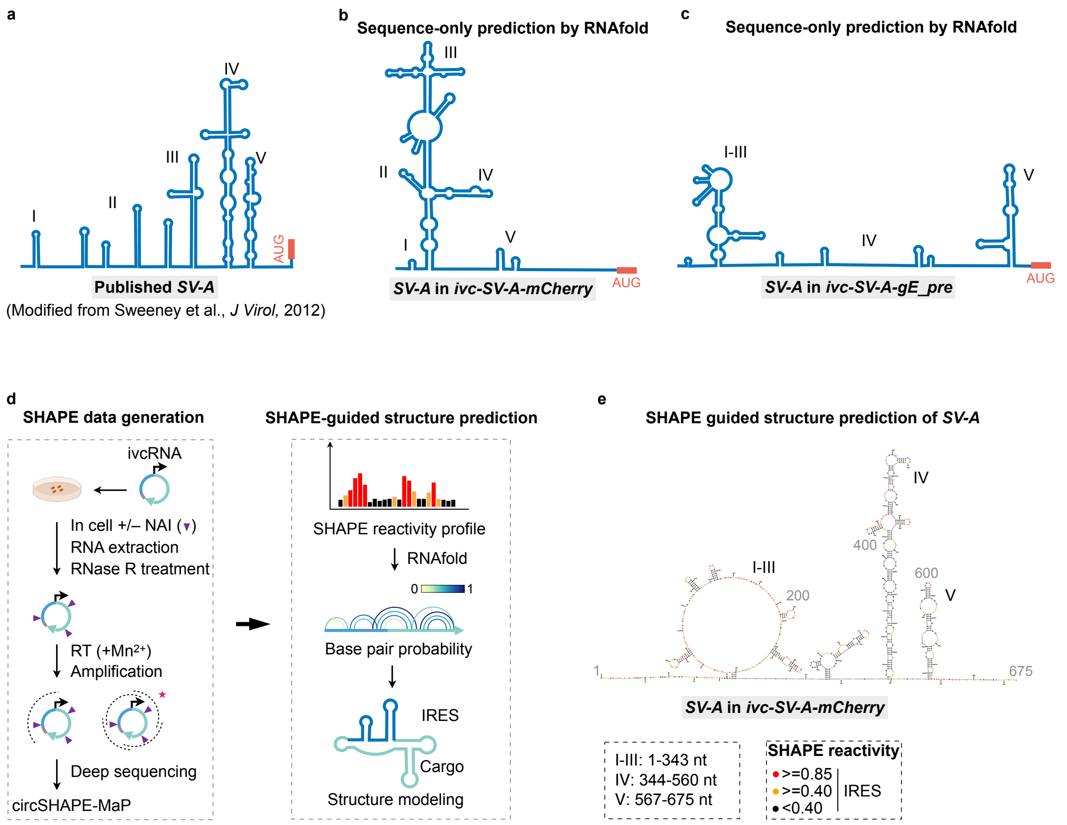

Supplementary Fig. S3: Secondary structure modeling of ivcRNAs based on sequence-only or SHAPE-guided prediction.

a, Schematic of published secondary structure model of *SV-A* (modified from Sweeney et al., J Virol, 2012).[11]

b-c, Secondary structure model of *SV-A* in *ivc-SV-A-mCherry* (**b**) and *ivc-SV-A-gE_pre* (**c**), based on sequence-only prediction.

d, Illustration of experimental procedure and computational pipeline for in cell circSHAPE-MaP. NAI, 2-methylnicotinic acid imidazoline; RT, reverse transcription. See also Supplementary Methods for details.

e, Secondary structure of *SV-A* in *ivc-SV-A-mCherry* was modeled with in cell SHAPE reactivities. SHAPE reactivity in IRES displays with different color, as detailed by black showing no/low SHAPE reactivity with a normalized value below 0.4, orange showing moderate SHAPE reactivity with a normalized value between 0.4 and 0.85 and red showing high SHAPE reactivity with a normalized value above 0.85.

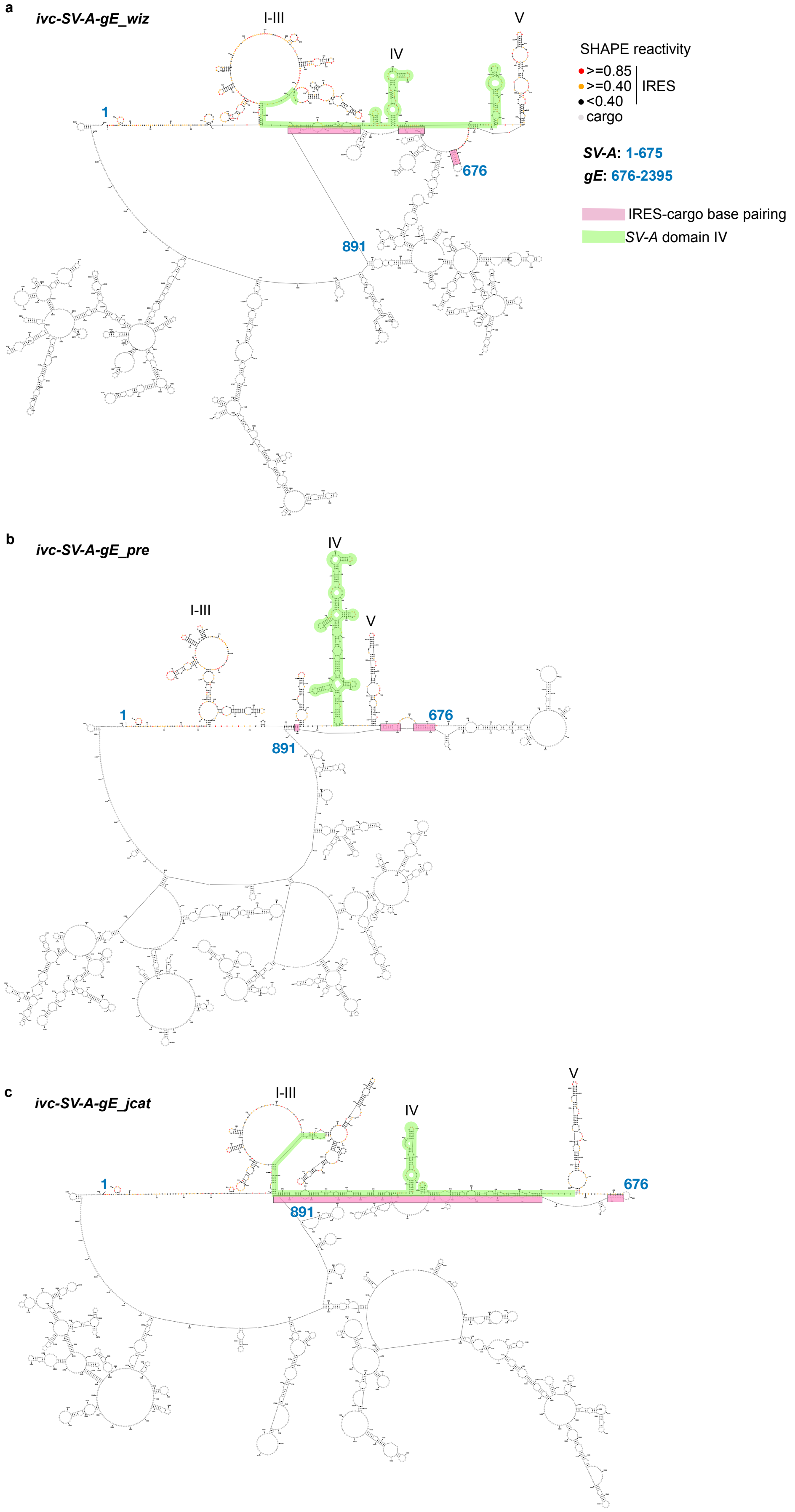
a
ivc-SV-A-gE_wiz
I-III
IV
V
1
676
891
SHAPE reactivity
>=0.85
>=0.40
<0.40
IRES
cargo
SV-A: 1-675
gE: 676-2395
IRES-cargo base pairing
SV-A domain IV
b
ivc-SV-A-gE_pre
IV
V
I-III
1
676
891
c
ivc-SV-A-gE_jcat
I-III
IV
V
1
676
891

Supplementary Fig. S4: Interaction between *SV-A* and *gE* sequences based on circSHAPE-guided secondary structures.

a-c, IRES-cargo interaction as displayed by secondary structure of ivcRNAs. Secondary structure of *ivc-SV-A-gE_wiz* (**a**), *ivc-SV-A-gE_pre* (**b**) and *ivc-SV-A-gE_jcat* (**c**) was modeled with in cell SHAPE reactivities. Base pairs between *SV-A* domain IV and *gE* sequences are highlighted in pink, and *SV-A* domain IV is highlighted in green. SHAPE reactivity in IRES displays with different color, as detailed by black showing no/low SHAPE reactivity with a normalized value below 0.4, orange showing moderate SHAPE reactivity with a normalized value between 0.4 and 0.85 and red showing high SHAPE reactivity with a normalized value above 0.85. The cargo sequences are colored in gray.

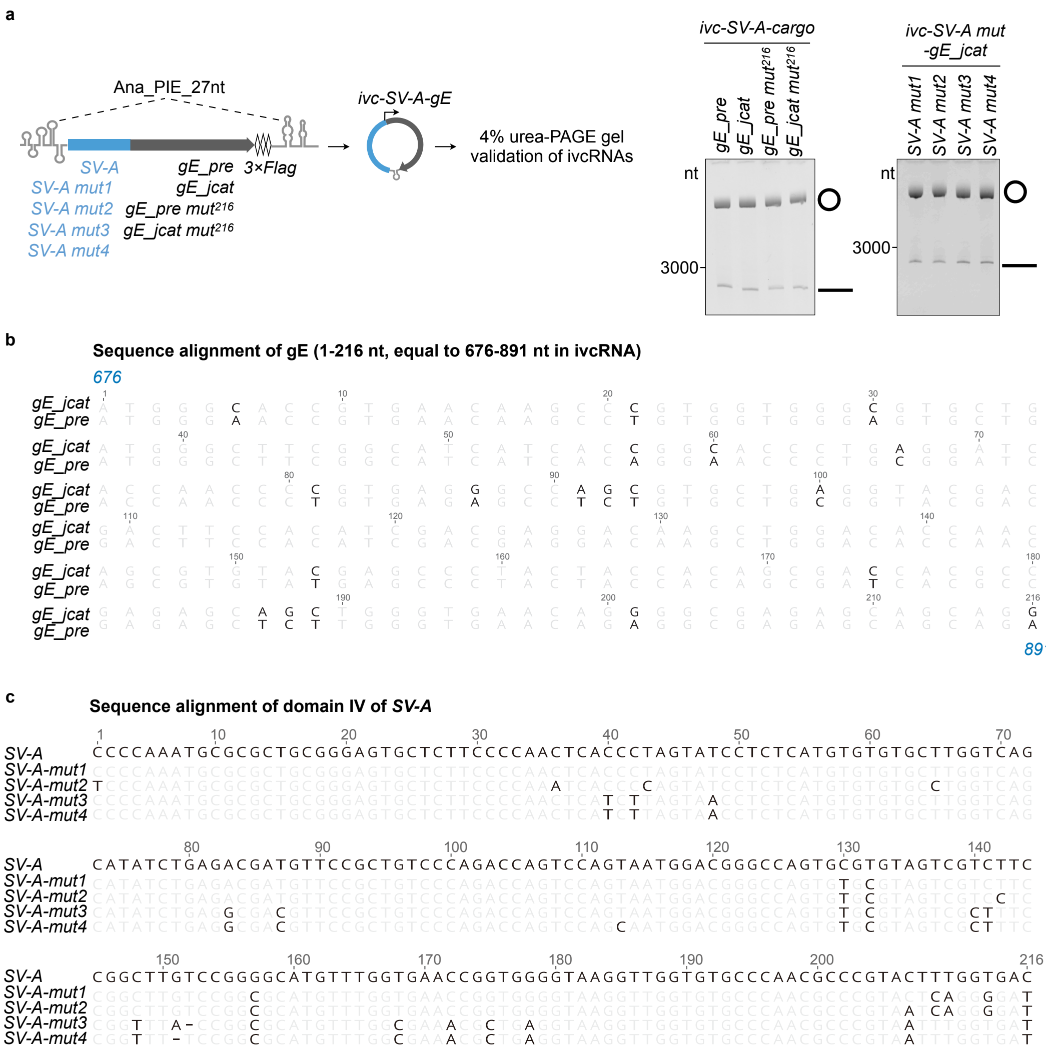

Supplementary Fig. S5: Characterization of *SV-A*-driven gE expression in engineered ivcRNAs

a, Schematic of engineered *SV-A* IRES and *gE* cargo for translation in ivcRNAs. Distinct ivcRNAs were *in vitro* synthesized using Ana_PIE_27nt system and then were validated by denaturing urea-PAGE gel (right). Circular RNA is indicated by black circle, nicked RNA indicated by black line. mut, mutant.

b, Sequence alignment of *gE_pre* and *gE_jcat* sequences at 1-216 nt. The 19 inconsistent nucleotides are shown in black.

c, Sequence alignment of four mutations of *SV-A* domain IV. The inconsistent nucleotides compared with *SV-A* are shown in black. mut, mutant.

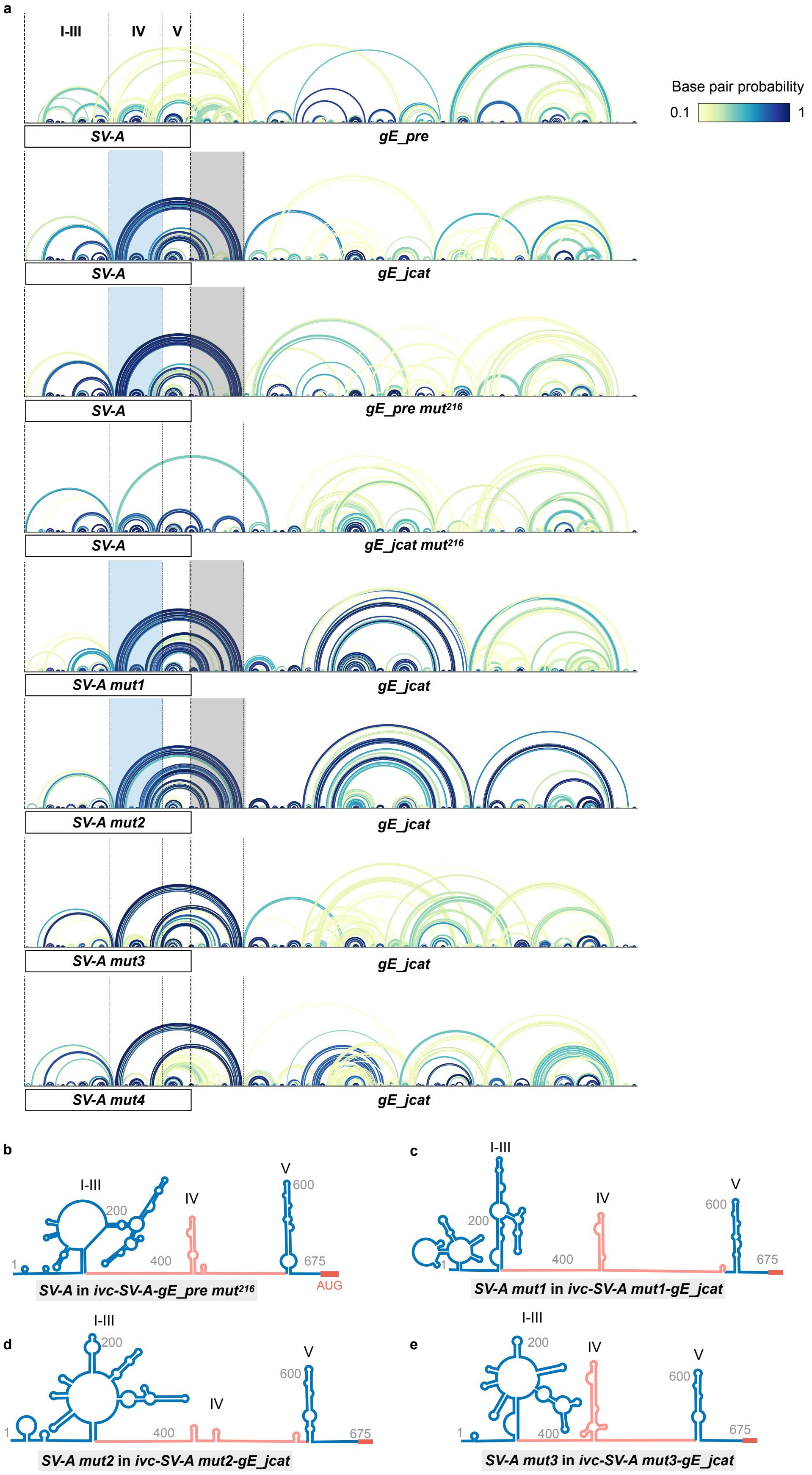
a
I-III
IV
V
Base pair probability
0.1
1
SV-A
gE_pre
SV-A
gE_jcat
SV-A
gE_pre mut216
SV-A
gE_jcat mut216
SV-A mut1
gE_jcat
SV-A mut2
gE_jcat
SV-A mut3
gE_jcat
SV-A mut4
gE_jcat
b
I-III
IV
V
SV-A in ivc-SV-A-gE_pre mut216
AUG
c
SV-A mut1 in ivc-SV-A mut1-gE_jcat
d
SV-A mut2 in ivc-SV-A mut2-gE_jcat
e
SV-A mut3 in ivc-SV-A mut3-gE_jcat
1
200
400
600
675

Supplementary Fig. S6: Alternation of interactions between IRES and cargo by manipulating *SV-A* and *gE* sequences

a, Base pair probability was obtained by RNAfold based on in cell SHAPE reactivities. Compared to *ivc-SV-A-gE_pre*, the base pair probability was increased in *ivc-SV-A-gE_pre mut^{216}*, *ivc-SV-A mut1-gE_jcat* and *ivc-SV-A mut2-gE_jcat*. By contrast, high base pair probability between *SV-A* and *gE_jcat* sequence was decreased after mutated *gE_jcat* sequence (*gE_jcat mut^{216}*) and mutated *SV-A* domain IV (*SV-A mut3* and *SV-A mut4*). The dotted line separates the region of *SV-A* domains (I-III, IV and V). The blue shadow and gray shadow indicate the IRES region (blue) and cargo region (gray) which interacted with each other with high probability.

b-e, Secondary structure of *SV-A* was modeled by circSHAPE-guided prediction in *ivc-SV-A-gE_pre mut^{216}* (**b**), *ivc-SV-A mut1-gE_jcat* (**c**), *ivc-SV-A mut2-gE_jcat* (**d**) and *ivc-SV-A mut3-gE_jcat* (**e**). The domain IV was marked as magenta. mut, mutant.

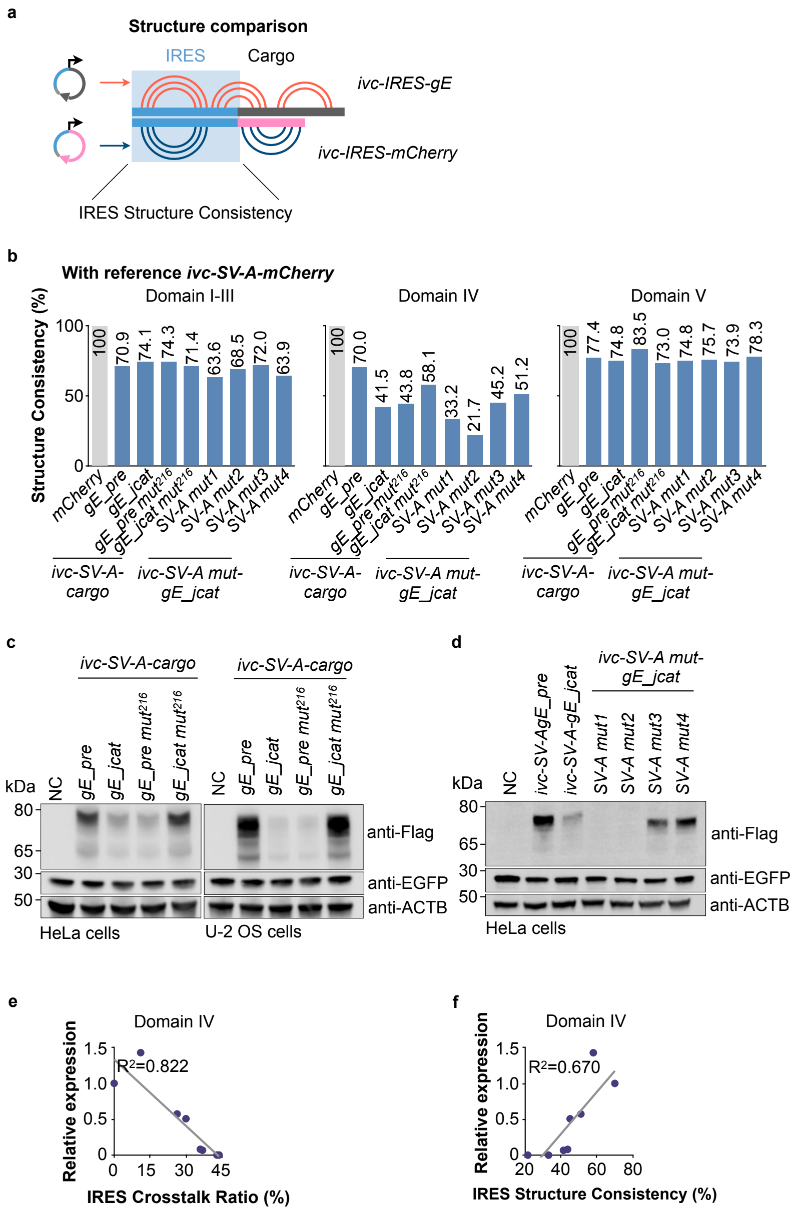

Supplementary Fig. S7: Disrupting interactions of IRES-cargo recovered gE expression in ivcRNAs

a, Schematic of the strategy for computational analyses of IRES Structure Consistency in particular ivcRNA compared to *ivc-SV-A-mCherry*.

b, IRES Structure Consistency of *SV-A* domains in engineered *ivc-SV-A-gEs* was measured with reference in *ivc-SV-A-mCherry*. Domains of *SV-A* were separated into three portions (domain I-III, IV and V). mut, mutant.

c-d, Restoring gE protein expression by disrupting IRES-cargo base pairing. WB

detected the gE protein expression from engineered *gE* driven by *SV-A* in HeLa and U-2 OS cells (**c**), and *gE_jcat* driven by engineered *SV-A* in HeLa cells (**d**). NC, negative control, only transfected with *EGFP* mRNA. mut, mutant.

e-f, The structural integrity of *SV-A* IRES correlated with cargo expression. A negative correlation was observed between the IRES domain IV Crosstalk Ratio and protein expression (**e**), while a positive correlation between the IRES domain IV Structure Consistency and protein expression (**f**). Relative gE expression levels were quantified from three independent biological replicates in 293FT cells; see also Supplementary Table S4 for detailed values of relative gE expression levels.

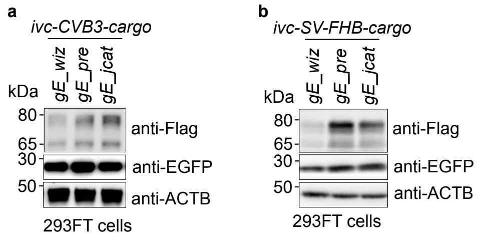

Supplementary Fig. S8: The gE protein expression from codon-optimized *gE* variants driven by *CVB3* and *SV-FHB* IRESs in ivcRNAs

a-b, Distinct gE protein expression levels were observed from different combinations of distinct cargo sequences (*gE_wiz, gE_pre or gE_jcat*) and two IRESs (*CVB3* or *SV-FHB*). The gE protein was indicated to be expressed at a higher level in a *CVB3*-driven ivcRNA with *gE_jcat* than that with *gE_wiz* or *gE_pre*, while gE protein was highly expressed in a *SV-FHB* driven ivcRNA with *gE_pre* than that with *gE_wiz* or *gE_jcat*.

Supplementary Table S1. Sequences of IRES elements and ivcRNAs used in this study.

Supplementary Table S2. SHAPE Primers for IRES and *SV-A-gE* variants in ivcRNA, and primers for qPCR.

Supplementary Table S3. The corresponding numerical data of SHAPE reactivity of the *SV-A* and mutated *SV-A* IRES within different ivcRNAs.

Supplementary Table S4. Statistical data of gE expression, IRES Crosstalk Ratio and IRES Structure Consistency. Relative gE expression levels were quantified from three independent biological replicates using Western blotting in 293FT cells.

ivcRNAs	Relative expression (*ivc-SV-A-gE_pre* as reference)	IRES Crosstalk Ratio of domain IV	IRES Structure Consistency of domain IV (*ivc-SV-A-mCherry* as reference)
ivc-SV-A-gE_pre	1	0	0.7005
ivc-SV-A-gE_jcat	0.06798967	0.3687	0.4147
ivc-SV-A-gE_pre mut^{216}	0.081406912	0.3594	0.4378
ivc-SV-A-gE_jcat mut^{216}	1.427332276	0.1106	0.5806
ivc-SV-A mut1-gE_jcat	0.001194348	0.4378	0.3318
ivc-SV-A mut2-gE_jcat	0.001212792	0.4286	0.2166
ivc-SV-A mut3-gE_jcat	0.50736213	0.2995	0.4516
ivc-SV-A mut4-gE_jcat	0.573608904	0.2627	0.5115

References

1 Zhang, Z. *et al.* Identification of B-cell epitopes on structural proteins VP1 and VP2 of Senecavirus A and development of a multi-epitope recombinant protein vaccine. *Virology* **582**, 48-56 (2023).

2 Karbalaei Zadeh Babaki, M., Soleimanpour, S. & Rezaee, S. A. Antigen 85 complex as a powerful Mycobacterium tuberculosis immunogene: Biology, immune-pathogenicity, applications in diagnosis, and vaccine design. *Microb Pathog* **112**, 20-29 (2017).

3 Monslow, M. A. *et al.* Immunogenicity generated by mRNA vaccine encoding VZV gE antigen is comparable to adjuvanted subunit vaccine and better than live attenuated vaccine in nonhuman primates. *Vaccine* **38**, 5793-5802 (2020).

4 Grote, A. *et al.* JCat:: a novel tool to adapt codon usage of a target gene to its potential expression host. *Nucleic Acids Research* **33**, W526-W531 (2005).

5 Wesselhoeft, R. A., Kowalski, P. S. & Anderson, D. G. Engineering circular RNA for potent and stable translation in eukaryotic cells. *Nat Commun* **9**, 2629 (2018).

6 Spitale, R. C. *et al.* Structural imprints in vivo decode RNA regulatory mechanisms. *Nature* **519** (2015).

7 Smola, M. J., Rice, G. M., Busan, S., Siegfried, N. A. & Weeks, K. M. Selective 2'-hydroxyl acylation analyzed by primer extension and mutational profiling (SHAPE-MaP) for direct, versatile and accurate RNA structure analysis. *Nature protocols* **10**, 1643-1669 (2015).

8 Liu, C.-X. *et al.* Structure and Degradation of Circular RNAs Regulate PKR Activation in Innate Immunity. *Cell* **177**, 865-880 e821 (2019).

9 Guo, S. K., Nan, F., Liu, C. X., Yang, L. & Chen, L. L. Mapping circular RNA structures in living cells by SHAPE-MaP. *Methods* **196**, 47-55 (2021).

10 Lorenz, R. *et al.* ViennaRNA Package 2.0. *Algorithms Mol Biol* **6**, 26 (2011).

11 Sweeney, T. R., Dhote, V., Yu, Y. & Hellen, C. U. A distinct class of internal ribosomal entry site in members of the Kobuvirus and proposed Salivirus and Paraturdivirus genera of the Picornaviridae. *J Virol* **86**, 1468-1486 (2012).
